# Allostatic load predicts racial disparities in intracerebral hemorrhage cognitive outcomes

**DOI:** 10.1038/s41598-022-20987-x

**Published:** 2022-10-03

**Authors:** Jennifer Harris, Amelia Boehme, Luisa Chan, Harmon Moats, Rachelle Dugue, Chigozirim Izeogu, Marykay A. Pavol, Imama A. Naqvi, Olajide Williams, Randolph S. Marshall

**Affiliations:** 1 Division of Stroke and Cerebrovascular Disease, Department of Neurology, Cedars-Sinai Medical Center, 127 S. San Vincente Blvd. #A6600, Los Angeles, CA USA; 2 Division of Stroke and Cerebrovascular Disease, Department of Neurology, Columbia University Medical Center, New York, NY USA; 3 Division of Neurology Clinical Outcomes Research and Population Sciences (Neuro CORPS), Department of Neurology, Columbia University Medical Center, New York, NY USA; 4 Department of Population Health, NYU Grossman School of Medicine, New York, NY USA

**Keywords:** Neuroscience, Biomarkers, Diseases, Health care, Medical research, Neurology, Risk factors

## Abstract

A large portion of stroke disparities remains unexplained, even after adjusting for demographic, comorbidity, and health care access variables. There is a critical need to close this knowledge gap by investigating novel factors that may contribute to stroke disparities. Allostatic load (AL) is the lifetime adverse physiologic impact of needing to adjust to socially structured stressors such as racism. AL has been shown to increase health vulnerability and worsen outcomes in marginalized populations. We sought to assess the differential impact of AL on cognitive outcomes post intracerebral hemorrhage (ICH) across race-ethnicity. The Intracerebral Hemorrhage Outcomes Project (ICHOP) prospectively collected data from patients presenting to Columbia Medical Center with ICH from 3/2009 to 5/2016. Data included demographics, stroke scores, labs, complications, neuroimaging, medical history, and discharge data. Five markers of AL (HbA1c, WBC, SBP, HR, ALB) were obtained. An AL score was generated by summing the elements in each patient that fell outside normal ranges, with AL score ranging 0–5. A linear regression model, adjusted for stroke severity and ICH volumes, was used to evaluate the relationship between AL and Modified Telephone Interview for Cognitive Status (TICS-m) at discharge, stratified by race-ethnicity. Among 248 white, 195 black, and 261 Hispanic ICH patients, neither mean AL nor mean TICS differed by race/ethnicity (p = 0.51, p = 0.79 respectively). In the overall cohort AL did not predict TICS at discharge (Beta -1.0, SE 1.1, p = 0.353). In Whites (beta 1.18, SE 2.5, p = 0.646) and Hispanics (beta -0.95, SE 1.6, p = 0.552) AL was not associated with TICS at discharge. In Black patients, higher AL was associated with a decrease in TICS at discharge (beta -3.2, SE 1.5, p = 0.049). AL is an important determinant of post ICH outcomes for certain minority populations. AL may explain some of the unexplained health disparities in stroke populations.

## Introduction

Health disparities have emerged as one of the great challenges to our health care system and a critical concern for the health of our U.S. population. The most dramatic disparities are seen in cardiovascular disease (CVD) pertaining to stroke outcomes including mortality and disability, as well as stroke risk. Race disparities in stroke outcomes in particular are widely reported in the literature. Whereas overall stroke rates in the U.S. have declined over past decades, stroke mortality rates in nonwhites (predominantly Non-Hispanic (NH) Black individuals) have remained substantially higher than in NH Whites^1^. Relative differences in stroke incidence also persist, with nearly a threefold increased risk between NH Black and NH White people among those < 55 years of age and a twofold increased risk in those > 55 years of age^2^. In the Northern Manhattan Study, among 3298 community-based participants, nonwhites had a nearly twofold increased stroke risk compared to Whites after accounting for socio-demographic characteristics; the risk for Hispanics was nearly identical^3^. Post-stroke disability has also been shown to vary by race, with post-stroke functional changes, including cognition, 33% higher in NH Black vs NH White patients, adjusting for socio-demographics (age, sex, marital status, education, income, insurance status, and social network size), comorbidities, hospitalization factors, post-acute care, and 90-day readmissions^4^. Despite accumulating evidence for disparities, data from the REasons for Geographic And Racial Differences in Stroke (REGARDS) study suggest that the prevalence of stroke risk factors, particularly hypertension and diabetes, while clearly higher among NH Black people, account for only 40% of the Black-White disparities in stroke incidence. The reasons for the remaining 60% are elusive^5^.

Since a large proportion of stroke disparities remains unexplained, there is a critical need to look for non-traditional risk factors that might contribute to those differences. One such novel potential target is Allostatic Load (AL), which is defined as the cumulative life impact of needing to adjust to chronic, repetitive stressors outside the normal range. Allostasis in the short term may be advantageous (e.g. the “fight or flight” response), but if stressors continue repeatedly or persist chronically there is a cost to the body and the brain as systems become dysregulated and shift their operating ranges to adapt to the stressors. Persistent or chronically repeated allostasis creates the *Allostatic Load*^6,7^. As a composite physiological marker, AL can be measured by summing physiological variables that reflect primary stressor-induced activation of endocrine and sympathetic nervous systems and secondary dysregulation of the cardiovascular and inflammatory systems^8^. A major contributor to AL is chronic exposure to, and having to cope with, socially structured stressors (SSS) such as racial discrimination^9^. AL has been shown to contribute to poor health outcomes in race-ethnicity and sex-gender minority populations ^10,11^ including for CVD and late life cognitive impairment^12,13^, but has never been applied to cognitive outcomes after stroke. We sought to assess the differential impact of AL on cognitive outcomes after intracerebral hemorrhage (ICH) across race-ethnicity using a comprehensive clinical research database from a large, urban tertiary stroke center. We hypothesized that Black patients would be more vulnerable to AL effects on post-ICH cognitive outcomes than White and Hispanic patients.

## Method

The Intracerebral Hemorrhage Outcomes Project (ICHOP) at Columbia University prospectively collected data from all intracerebral hemorrhage (ICH) patients presenting to the hospital with a diagnosis of ICH from March 2009 to May 2016. The ICHOP study methods have been previously described in detail^14^. The study was approved by the Institutional Review Board (IRB) committee at our institution, and written informed consent was obtained from all patients (or respective legal guardians when the patient was unable to provide consent) participating in the study. All subjects were screened by treating physicians. Subjects with non-spontaneous ICH were excluded. All participants or designated proxies underwent a standardized data collection protocol, which included a personal interview and medical chart abstraction. Data included demographics, stroke scores, labs, neurological deterioration, hospital complications, neuroimaging, medical history, and discharge data. Data was extracted from the EHR by trained research assistants. Head CT imaging of each patient was assessed by four of the treating neurointensivists and the attending neurosurgeon. Radiographic variables assessed included hematoma volume and location, and presence of intraventricular extension. The presumed etiology of each ICH was determined by consensus among the treating neurointensivists during a weekly meeting, based on a combination of demographic/clinical data and radiographic appearance. Admission hematoma volumes were measured by two ICHOP investigators blinded to patient outcome using MIPAV software package (V.4.3, National Institutes of Health, Bethesda, Maryland, USA) and independent observer results were averaged. Cognitive scores were obtained by trained research assistants who were blinded to clinical data. Race and ethnicity were self-reported. Baseline demographic and medical history data included age, sex, race, ethnicity, and medical comorbidities.

### Inclusion/exclusion criteria

All patients 18 and older who had ICH were included in ICHOP. We excluded patients who did not identify as either NH-white, NH-Black, or Hispanic. We excluded patients with ICH due to trauma or cancer. Subarachnoid hemorrhage or hemorrhagic conversion of ischemic stroke were not included.

### Exposure of interest

The exposure of interest was AL, calculated from clinical and laboratory measures which were available at baseline at time of admission from the ICHOP database and are standard of care stroke admission labs. Five markers of AL (hemoglobin A1C (HbA1c), systolic blood pressure (SBP), heart rate (HR), albumin (ALB) and white blood count (WBC) were used. The upper limit of normal lab values was used to assign AL scores for each laboratory measure. For SBP, an abnormal value was defined as > 140 mmHg and for HR, > 100 bpm. Past medical history of hypertension or diabetes were incorporated into the AL score if SBP or A1c were not available (e.g. either SPB > 140 or history of hypertension would count as a positive AL for abnormal blood pressure). Each component of the AL score was dichotomized by within normal range vs. not within normal range. One point was assigned to each AL marker that was out of normal range. AL score was generated by summing the number of markers in each patient that fell outside normal clinical ranges. AL score could thus range from 0 to 5.

### Outcome of interest

The primary outcome measure was the Modified Telephone Interview for Cognitive Status (TICS-m) at discharge. The TICS-m is a validated global mental status test that can be administered over the telephone. Cognitive domains measured by the TICS-m include orientation, concentration, short-term and delayed recall, language, praxis, and mathematical skills. It includes 12 items, with each individual item score summed to obtain the TICS-m total score. The TICS-m total score provides a measure of global cognitive functioning and can be used to monitor changes in cognitive functioning over time^15^. Although it is possible to calculate z-scores for TICS-m results, our sample included subjects of different race-ethnicities. Because there are no published norms for TICS for Black or Hispanic populations, we opted to use the raw scores and adjust for race-ethnicity and age in our model to achieve a more accurate analysis.

### Statistical analyses

Demographic and clinical characteristics were compared using frequency and percent for categorical variables and mean/standard deviation or median/range for continuous variables. We used a linear regression model to evaluate the relationship between the exposure of interest, AL, and the outcome of interest, TICS at discharge, in the entire population. To evaluate whether there was effect measure modification by race/ethnicity (our study hypothesis), we stratified the population by race-ethnicity and ran the linear regression model to evaluate AL on TICS within each race/ethnic group. The ICH score, a validated clinical grading scale that includes Glasgow Coma Scale, age, ICH volume, ICH location and presence of intraventricular blood, was used to adjust for clinical and radiographic severity.

### Ethics approval

This retrospective chart review study involving human participants was in accordance with the ethical standards of the institutional and national research committee and with the 1964 Helsinki Declaration and its later amendments or comparable ethical standards. The Human Investigation Committee (IRB) of Columbia University approved this study.

## Results

From a total cohort of 704 there were 248 White, 195 Black, and 261 Hispanic ICH patients for whom data were analyzed. In the cohort, 154 (21.8%) died at discharge. These included 23% of White, 21% of Black and 20% of Hispanic patients. Average age of the cohort was 63.5 (SD 20.8) with 53.7% men. There was no significant difference among the groups in regard to history of diabetes and smoking history. Baseline characteristics are shown in Table 1. Neither mean AL nor mean TICS-m differed by race/ethnicity (p = 0.51, p = 0.79 respectively). Of note, all TICS-m scores fell below published thresholds for normal performance of ≤ 31^15,16^, indicating that our sample as a whole had cognitive impairment. Examination of the data revealed no evidence of non-normality or outliers. Average values and the interquartile range did not indicate any floor or ceiling effect.

**Table 1:** Comparison of AL variables between White, Black and Hispanic patients.

	White (N = 248)	Black (N = 195)	Hispanic (N = 261)	p-value
Age, median (range)	71 (18–97)	60 (20–100)	63 (18–97)	0.006
Male sex, %	56.45	50.26	50.57	0.243
Smoking, %	10.5	23.8	9.6	0.000
High A1C, %	32.26	37.44	44.06	0.081
High sbp, %	61.29	69.23	69.73	0.377
High WBC, %	36.74	24.44	29.63	0.028
High HR, %	67.34	67.69	67.82	0.982
Abnormal alb, %	63.31	64.62	62.07	0.592
Allostatic load, mean (SD)	4.1 (1.7)	4.4 (1.6)	4.5 (1.6)	0.505
Allostatic load, median (IQR)	4 (3–5)	4 (4–5)	4 (4–5)	
TICS, median (IQR)	24 (3–33)	23 (12–30)	21 (3–29)	0.788

Our primary aim was to exam the relationship between AL and cognition within each race-ethnicity group. In the overall cohort AL did not predict TICS-m at discharge (Beta −1.0, SE 1.1, p = 0.353). In Whites (beta 1.18, SE 2.5, p = 0.646) and Hispanics (beta −0.95, SE 1.6, p = 0.552) AL was not associated with TICS at discharge. In Black patients, higher AL was associated with a decrease in TICS-m at discharge (beta −3.2, SE 1.5, p = 0.049) (Table 2). Adjusting for ICH severity using the ICH Score did not significantly change the results**.**

**Table 2:** Comparison of AL load and associated TICS at discharge between White, Black and Hispanic patients.

	Beta (SE)	p-value
AL in entire Cohort	−1.0 (1.1)	0.353
AL within white participants	1.18 (2.5)	0.646
AL within black participants	−3.2 (1.5)	0.049
AL within Hispanic participants	−0.95 (1.6)	0.552

## Discussion

A disturbingly large portion of stroke disparities remains unexplained, even after accounting for a plethora of demographic, comorbidity, risk factor, and health care access variables^5^. Cognitive outcomes post-stroke have been reported to vary by race^17^, but the reasons for this disparity has remained elusive. In the current study, we demonstrated that, although *mean* AL score did not differ among Black, White and Hispanic patients, the impact of AL on cognition varied significantly by race-ethnicity. We found that for Black patients, higher AL was associated with worse cognitive outcome such that for every 1-point increase in AL, there was a 3-point decrease in TICS-m score at discharge. For Whites and Hispanics, by contrast, AL score did correlate at all with TICS-m at discharge, suggesting that AL affects cognitive outcomes for certain minority populations differentially. To our knowledge this is the first study to investigate AL and its relation to a post stroke outcome.

Several lines of evidence link psychosocial stress to dysregulation of endocrine, autonomic nervous system and cardiovascular systems. Sympathetic nervous system overdrive results in catecholamine production, increased inflammation, and lipid dysregulation, pushing systems to regulate outside of their normal bounds^18^. Over time, chronic stress produces persistent allostasis, resulting in an “allostatic load” that is measurable and impactful, as in our current population.

There is growing evidence connecting stress to the development and progression of CVD. For example, a large population-based screening and intervention program for CVD risk factors which included 13,609 individuals, showed that over two decades high perceived stress was independently associated with CVD and specifically, fatal stroke (relative risk 2.0, 95% CI 1.1–3.9)^19^. A meta-analysis of six prospective observational cohort studies that measured perceived stress and incident coronary heart disease at least 6 months later, estimated a cumulative risk ratio of 1.27 (95% CI 1.12–1.45) for the association between high perceived stress and incident coronary heart disease^20^. Another large study, which consisted of 24,767 individuals from around the world, supported the importance of multiple psychosocial factors in relation to atherosclerotic risk. The study found that persistent psychological stressors linked to finances, home, work and life events were associated with a twofold increase in risk of acute myocardial infarction^21^.

Psychosocial stress may also contribute to the Black–White disparities seen in CVD.

There is mounting evidence that social determinants of health (SDOH) including neighborhood factors, unemployment, financial difficulties, childhood adversity, racial and ethnic discrimination and numerous other stressors affect cardiovascular health, morbidity and mortality beyond the effects of access to healthcare^22^. Duru et al. utilizing National Health and Nutrition Examination (NHANES) found that measures of AL were significantly higher in Black than in White participants, such that a higher AL score associated with black participants corresponded to at least a 2.5-fold greater mortality compared to Whites^23^.

Our study advanced the AL hypothesis by showing an association between AL and TICS-m for Black, but not for White or Hispanic patients, even though there was no difference in group mean AL scores by race/ethnicity. We interpret this finding to be in support of our hypothesis that, while chronic stress is a part of everyone’s life, certain populations may be exposed to more chronic stress and/or have less buffering protection afforded by stable, responsive relationships and supportive environments, which predisposes them to “toxic stress” and its long-lasting effects on AL^24^.

Data such as these support our finding that AL differentially affects certain minority populations. What remains to be seen is whether the AL effect can be mitigated or reduced, as shown in a study by Brody et al. in which AL impact appeared to be lessened among young adults with a positive racial identity^25^. A modifiable target to mitigate the effects of AL could potentially reduce disparities in stroke outcomes.

Several limitations of this study must be acknowledged. First, this was a single center, retrospective study of ICH only. Results will need to be validated prospectively and in other stroke subtypes. Second, our exposure measures were limited to the AL variables that were available within the ICHOP data base, collected at the time of stroke. Some of the values could have been influenced by the physiological stress of the stroke itself and primary markers of stress such as cortisol, dehydroepiandrosterone, epinephrine and norepinephrine were not available. This limitation was partially mitigated by adjusting for clinical and radiographic severity with the ICH score, which did not attenuate the magnitude of the relationship between AL score and TICS-m. In addition, there was a high prevalence of some AL measures such as high SBP and diabetes across the whole population, which reduced the variance of AL scores, making group mean differences in AL score more difficult to identify. Finally, we lacked a measure of depression, which can influence cognitive scores. Whereas many more AL variables have been reported in the literature than the ones used in this investigation, other studies have calculated AL in a variety of ways and shown similar results^8^. Despite the limitations, our AL measure, created a priori based on the existing data base, yielded positive results. Further prospective investigation with additional AL variables is needed to validate our results in ICH and other stroke subtypes.

In conclusion, our study showed that AL predicted cognitive scores at discharge for NH Black, but not Hispanic or White patients, suggesting that AL is an important determinant of post ICH outcomes for certain minority populations. AL may account for some of the unexplained health disparities in stroke populations. Moving forward, it will be valuable to identify how AL may mediate the relationship between socially structured stressors and stroke outcomes, with a goal of identifying potential interventions at the socio-behavioral and policy level that might mitigate disparities in stroke outcome.

## Data Availability

Data used for this study are available from the corresponding author upon reasonable request.

## Abbreviations

CVD: Cardiovascular disease
NH: Non-Hispanic
AL: Allostatic load
SSS: Socially structured stressors
ICH: Intracerebral hemorrhage
ICHOP: Intracerebral Hemorrhage Outcomes Project
HbA1c: Hemoglobin A1C
SBP: Systolic blood pressure
HR: Heart rate
ALB: Albumin
WBC: White blood count
TICS: Telephone Interview for Cognitive Status
